# Explainable Image Quality Assessments in Teledermatological Photography

**DOI:** 10.1089/tmj.2022.0405

**Published:** 2023-08-29

**Authors:** Raluca Jalaboi, Ole Winther, Alfiia Galimzianova

**Affiliations:** ^1^ Department of Applied Mathematics and Computer Science, Technical University of Denmark, Lyngby, Denmark.; ^2^ Medable A/S, Copenhagen, Denmark.; ^3^ Pioneer Centre for AI, Copenhagen, Denmark.; ^4^ Bioinformatics Centre, Department of Biology, University of Copenhagen, Copenhagen, Denmark.; ^5^ Center for Genomic Medicine, Rigshospitalet, Copenhagen University Hospital, Copenhagen, Denmark.

**Keywords:** teledermatology, image quality, artificial intelligence, deep learning, explainability, telemedicine

## Abstract

**Background and Objectives:**

Image quality is a crucial factor in the effectiveness and efficiency of teledermatological consultations. However, up to 50% of images sent by patients have quality issues, thus increasing the time to diagnosis and treatment. An automated, easily deployable, explainable method for assessing image quality is necessary to improve the current teledermatological consultation flow. We introduce ImageQX, a convolutional neural network for image quality assessment with a learning mechanism for identifying the most common poor image quality explanations: bad framing, bad lighting, blur, low resolution, and distance issues.

**Methods:**

ImageQX was trained on 26,635 photographs and validated on 9,874 photographs, each annotated with image quality labels and poor image quality explanations by up to 12 board-certified dermatologists. The photographic images were taken between 2017 and 2019 using a mobile skin disease tracking application accessible worldwide.

**Results:**

Our method achieves expert-level performance for both image quality assessment and poor image quality explanation. For image quality assessment, ImageQX obtains a macro F1-score of 0.73 ± 0.01, which places it within standard deviation of the pairwise inter-rater F1-score of 0.77 ± 0.07. For poor image quality explanations, our method obtains F1-scores of between 0.37 ± 0.01 and 0.70 ± 0.01, similar to the inter-rater pairwise F1-score of between 0.24 ± 0.15 and 0.83 ± 0.06. Moreover, with a size of only 15 MB, ImageQX is easily deployable on mobile devices.

**Conclusion:**

With an image quality detection performance similar to that of dermatologists, incorporating ImageQX into the teledermatology flow can enable a better, faster flow for remote consultations.

## Introduction

Within the past 2 years, consumers facing teledermatological consultations have become much more common owing to the SARS CoV-2 (COVID-19) pandemic and associated worldwide isolation measures.^1^ Teledermatological consultations are carried out increasingly more often via teledermatology mobile applications that require patients to photograph their skin lesions using their mobile devices, such as smartphones and tablets, and send them to dermatologists who will then diagnose the depicted skin condition remotely.^2,3^ To achieve similar quality of care to an in-person consultation, high-quality images are paramount.^2,3^ However, this is rarely the case: up to 50% of patients send images taken under poor lighting conditions, that are not centered on the lesion, or that are blurry.^4,5^

When dealing with low-quality images, two main approaches exist: image denoising and image quality detection. Image denoising processes and reconstructs noisy images such that the noise is either reduced or entirely removed. Many denoising methods introduce new artifacts into the images or obfuscate characteristics critical for diagnosis.^6^ Therefore, in this article we focus on image quality detection. By detecting low-quality images directly on the patient's mobile device, we can instruct them to retake the picture in a way that improves the quality to an acceptable level. We can thus reduce the evaluation burden on dermatologists while at the same time reducing the time to diagnosis and treatment.

Several methods for image quality detection have been proposed in the literature. Kim and Lee introduce DeepIQ,^7^ a deep neural network that can identify noisy sections in an image, and compare the resulting noise maps with human assessments. Bianco et al propose DeepBIQ,^8^ a convolutional neural network for identifying low-quality images, and report near human-level results on smartphone photos from the LIVE In the Wild challenge dataset.^9^ Madhusudana et al develop CONTRIQUE,^10^ a contrastive deep learning system for creating generalizable representations using unlabeled image quality datasets. One common issue for all methods is the lack of a reference standard label, which limits both their training and validation rigor. Because of this reason, they often use unsupervised training methods and limit validation to qualitative assessment.

Within teledermatology, Vodrahalli et al proposed a classical machine learning image quality classifier.^5^ Their method provides patients with explanations for the quality assessments through automated classical computer vision methods for detecting blur, lighting, and zoom issues in an image. However, this method has several limitations: it cannot handle cases where only the background is blurry or with poor lighting, it cannot detect lesion framing issues, and it cannot discard images containing no skin.

The lack of explainability is regarded as one of the biggest obstacles toward the adoption of automated methods in medical practice.^11–13^ Gradient-based class activation maps (Grad-CAM)^14^ is the most common explainability method in medical computer vision owing to its ease of use, intuitive output, and low computational requirements. Grad-CAM creates CAMs on a given convolutional layer using the backpropagation gradients—the higher the gradient, the more important the region is to the final classification.

**Fig. 1.**
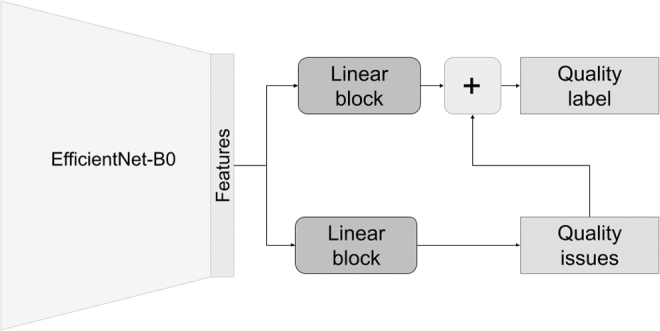
ImageQX network architecture. To facilitate deployment on mobile devices, we use the lightweight EfficientNet-B0 architecture as a feature extractor. A linear block, composed of a linear layer, batch normalization, and a dropout layer, is used to parse these features before predicting poor image quality explanations, that is, *bad framing*, *bad light*, *blurry*, *low resolution*, and *too far away*. Another similar linear block parses the image features and then concatenates them with the poor image quality explanations to predict the image quality label.

In this study, we introduce ImageQX, a convolutional neural network-based method for detecting image quality. Our novel approach uses image quality evaluations obtained from dermatologists in a teledermatology setting to learn the image quality required for a successful remote consultation. *Figure 1*illustrates the ImageQX architecture, which learns the image quality and its explanations in an end-to-end manner. ImageQX was trained and validated on 36,509 images collected using a skin lesion progression tracking mobile application. Images were labeled by up to 12 board-certified dermatologists. We evaluate the network performance with regard to the reference standard, and we obtain a macro F1-score of 0.73 for image quality assessment, with the per-explanation performance between 0.37 and 0.71. Moreover, ImageQX occupies only 15 MB, making it ideal for deploying on mobile devices as a prefiltering step during data collection.

## Methods

A total of 36,509 images were collected between 2017 and 2019, using Imagine,^15^ a skin disease tracking mobile application available worldwide. Self-reported user ages range between 18 and 80 years, and self-reported sex showing a distribution of 49% men, 47% women, and 4% other. Users span 146 countries, with images from Ukraine, United Kingdom, United States, Georgia, Russia, Albania, Kazakhstan, India, Denmark, South Africa, Bulgaria, and Israel making up 45% of the dataset. Images cover a wide variety of body parts. Self-reported body part tags show that faces, arms, elbows, legs, and groin comprise the majority of images. All data was anonymized a priori and did not involve human subjects. 45 CFR part 46 does not apply, and thus an independent ethics committee approval was not applicable for this research.

**Fig. 2.**
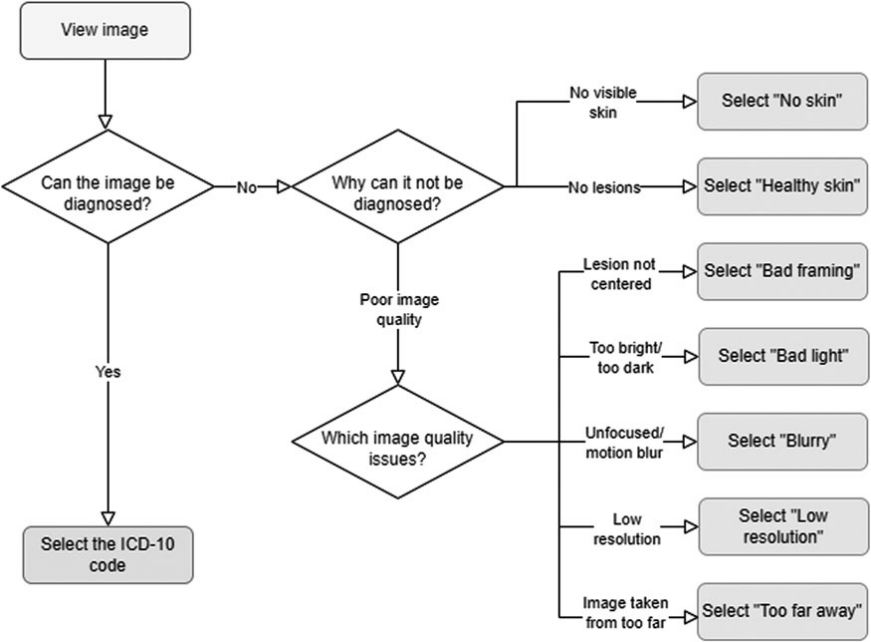
Labeling protocol for the ImageQX training and validation dataset. Dermatologists start by assessing whether or not the image can be diagnosed. If the image can be assessed, they diagnose it using an ICD-10 code. Otherwise, if there is no visible skin or if there are no visible lesions in the picture, the dermatologists discard the image as *no skin* or *healthy skin*, respectively. Finally, if the image cannot be evaluated because of poor quality, they select one of the five investigated poor image quality explanations.

**Fig. 3.**
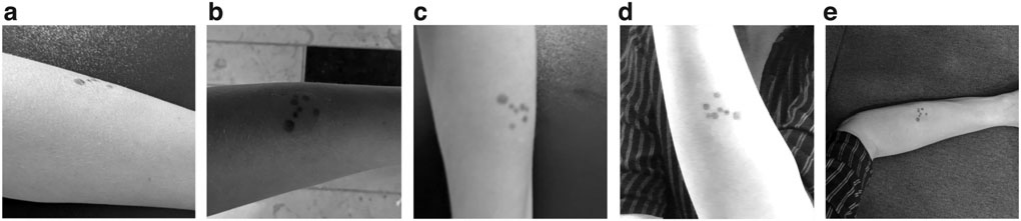
Illustration of poor image quality explanations that can be detected by ImageQX. **(a)** *Bad framing*: the image was not centered on the lesion. **(b)** *Bad light*: the lighting conditions in which the image was taken were too dark or too bright. **(c)** *Blurry*: the image is not focused on the lesion, masking out its details. **(d)** *Low resolution*: the image was taken with a low-resolution camera and few details can be discerned. **(e)** *Too far away*: few lesion details could be seen owing to the distance from the camera. Images courtesy of the authors.

Each image was evaluated by up to 12 board-certified dermatologists using an in-house labeling tool. Dermatologists diagnosed each image with an *International Classification of Diseases, 10th Revision* (ICD-10) code^16^ whenever a lesion was present in the image and was depicted with a sufficient quality, or alternatively with one of three nonlesion labels: *poor quality* when the image quality detracted from their ability to diagnose, *healthy skin* whenever no lesions were visible, or *no skin* for images that had no dermatological relevance. *Poor quality* images were additionally tagged with poor quality explanations: *bad framing* for images not centered on the lesion, *bad light* for images that are too bright or too dark, *blurry* for images suffering from motion blur or inadequate focus, *low resolution* for images taken with a low-resolution camera, or *too far away* for images where the picture was taken from afar and no details could be discerned. *Figure 2* outlines the protocol dermatologists followed when labeling the data, whereas *Figure 3* illustrates each poor image quality explanation included in the dataset.

We evaluate the performance of the raters and the network using sensitivity:


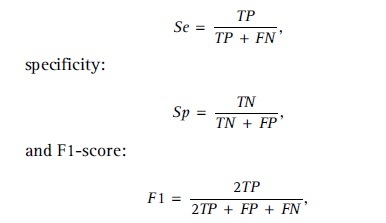


where *TP*, *FP*, and *FN* denote the true positives, false positives, and false negatives, respectively. The inter-rater pairwise F1-score is calculated as the average of all dermatologist pairs, where one dermatologist is considered the reference standard whereas the other is considered the prediction. For evaluating the network performance, we calculate the macro F1-score, that is, we average the F1-scores for each class.

During training, we parsed the dermatologist evaluations into four classes by merging all ICD-10 evaluations into the *lesion* class. We used plurality label fusion, that is, the class selected by most dermatologists, for defining the image quality class for each image. Alongside assessing whether the image can be evaluated, our proposed method also offers explanations to the poor quality images. To obtain the reference standard for poor image quality explanations, we chose to mark explanations as relevant if at least one dermatologist discarded an image with that explanation. *Table 1* provides the distribution of labels within the dataset, whereas *Table 2* details the distribution of poor image quality explanations. Higher agreement is achieved on *lesion* and *no skin*, whereas low agreement between raters can be seen for *healthy skin* and *poor quality*. Poor image quality explanations display low inter-rater agreements, with *blurry* being the only one achieving an inter-rater pairwise F1-score of >0.80.

**Table 1.** Distribution of Image Quality Labels Over the Training and Test Sets, Including the Pairwise Inter-Rater Agreement Calculated as the Pairwise F1-Score

CLASS	TRAIN IMAGE COUNT	TEST IMAGE COUNT	PAIRWISE TRAIN F1	PAIRWISE TEST F1
Lesion	17,534	4,803	0.86 ± 0.03	0.84 ± 0.08
No skin	461	265	0.93 ± 0.03	0.92 ± 0.04
Healthy skin	3,903	2,421	0.62 ± 0.10	0.65 ± 0.10
Poor quality	4,737	2,385	0.63 ± 0.08	0.67 ± 0.07
Mean	6658.75	2468.5	0.76 ± 0.06	0.77 ± 0.07

**Table 2.** Distribution of Poor Image Quality Explanations over the Training and Test Sets, Alongside the Pairwise Inter-Rater Agreement for Each Explanation, Calculated as the Pairwise F1-Score

REASON	TRAIN IMAGE COUNT	TEST IMAGE COUNT	PAIRWISE TRAIN F1	PAIRWISE TEST F1
Bad framing	1,947	982	0.26 ± 0.18	0.24 ± 0.15
Bad light	5,144	2,481	0.63 ± 0.07	0.65 ± 0.08
Blurry	5,499	2,640	0.81 ± 0.05	0.83 ± 0.06
Low resolution	3,965	1,907	0.33 ± 0.14	0.32 ± 0.14
Too far away	936	497	0.48 ± 0.16	0.51 ± 0.30
Mean	4372.75	2126.75	0.63 ± 0.15	0.64 ± 0.18

The ImageQX architecture is inspired by the DermX architecture introduced by Jalaboi et al to intrinsically learn the expert explanations, as illustrated in *Figure 1*.^17^ EfficientNet-B0 was used as the feature extractor to increase the image processing speed and reduce the network size.^18^ To increase the convergence speed, we used weights pretrained on the ImageNet dataset,^19^ made available by the Pytorch framework.^20^ Our network optimizes Equation (1) from Jalaboi et al^17^:


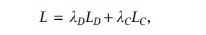


where *L_D_* is the categorical cross-entropy loss for the image quality label


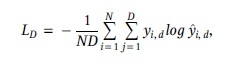


and *L_C_* is the binary cross-entropy loss for poor image quality explanations


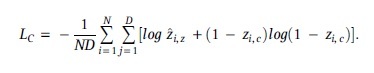


We set *λ_D_* = 1.0 and *λ_C_* = 5.0. To address the imbalance in image quality labels, we used class weighted training. Weights were set inversely proportional to frequency in training set, as follows:


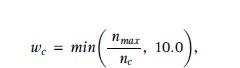


where *w_c_* is the weight associated with each sample in class *c*, *n_c_* is the number of samples in class *c*, and *n_max_* is the number of samples in the most common class. Class weights were clipped to 10.0 to avoid overfitting on small classes. This process resulted in 1.0, 10.0, 4.49, and 3.70 as weights for *lesion*, *no skin*, *healthy skin*, and *poor quality*, respectively. The network was trained for 39 epochs with the AdamW optimizer,^21^ cosine annealing with warm restarts,^22^ 64 U in each linear block, and 0.2 dropout. Five runs with identical hyperparameters were performed to estimate the standard deviation between training runs.

## Results

*Table 3* provides the image quality assessment performance, whereas *Table 4* provides the performance on each poor image quality explanation. The F1-scores for *healthy skin* and *poor quality* are within standard deviation of the inter-rater agreement, whereas for *lesion* and *no skin* the performance is slightly lower. The lower performance on *no skin* may be explained by the limited training data available. For poor image quality explanations, all F1-scores except for *blurry* are within standard deviation of the mean inter-rater agreement. The high specificity visible in both image quality assessment and in poor image quality explanation suggests that deploying this network on patient phones would not negatively impact the patient experience by rejecting high-quality images.

**Table 3.** ImageQX Performance on Image Quality Assessment over Five Training Runs (Mean ± Standard Deviation)

CLASS	SENSITIVITY	SPECIFICITY	F1-SCORE
Lesion	0.84 ± 0.03	0.78 ± 0.04	**0.82 ± 0.00**
No skin	0.76 ± 0.05	0.99 ± 0.00	0.74 ± 0.02
Healthy skin	0.61 ± 0.09	0.90 ± 0.02	**0.63 ± 0.04**
Poor quality	0.71 ± 0.02	0.93 ± 0.00	**0.74 ± 0.01**
Mean	0.73 ± 0.01	0.90 ± 0.01	**0.73 ± 0.01**

F1-scores in bold show the assessments where ImageQX reaches expert-level performance.

**Table 4.** ImageQX Performance on Poor Image Quality Explanation Performance over Five Training Runs (Mean ± Standard Deviation)

REASON	SENSITIVITY	SPECIFICITY	F1-SCORE
Bad framing	0.31 ± 0.01	0.96 ± 0.00	**0.37 ± 0.01**
Bad light	0.58 ± 0.02	0.90 ± 0.01	**0.61 ± 0.00**
Blurry	0.60 ± 0.02	0.95 ± 0.00	0.70 ± 0.01
Low resolution	0.47 ± 0.02	0.92 ± 0.01	**0.52 ± 0.01**
Too far away	0.35 ± 0.02	0.98 ± 0.00	**0.42 ± 0.02**
Mean	0.39 ± 0.01	0.95 ± 0.00	**0.45 ± 0.01**

F1-scores in bold show the explanations where ImageQX reaches expert-level performance.

**Fig. 4.**
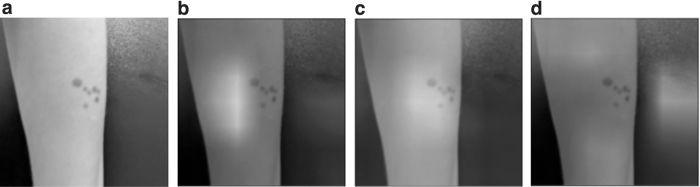
Grad-CAM attention maps for the *blurry* test image introduced in *Figure 3*. The image was correctly classified as *poor quality*. **(a)** Original *blurry* image. **(b)** Grad-CAM attention map for *bad light*. **(c)** Grad-CAM attention map for *blurry*. **(d)** Grad-CAM attention map for *low resolution*. When predicting *bad light*, ImageQX focuses on a slightly shaded part of the arm, whereas for *blurry* it highlights the lesion and its surrounding area. The *low-resolution* prediction is based on the edges of the arm and the background. Image courtesy of the authors. Grad-CAM, gradient-based class activation map.

*Figure 4* provides the Grad-CAM attention maps for each poor image quality explanation detected in a *blurry* image. ImageQX correctly detected *blurry* as one of the poor image quality explanations, focusing almost entirely on the skin area and paying more attention to the lesion. Two other explanations were also marked as present: *bad light* with a focus on a slightly shaded part of the arm, and *low resolution* that highlights the edges of the hand and a part of the background.

## Discussion

Our data-labeling process confirms the previously reported findings that poor image quality is a significant issue in teledermatology—around 20% of the images collected through the mobile application were labeled as poor quality by dermatologists. Dermatologists have low levels of agreement on which images are poor quality, with inter-rater F1-scores of 0.62 ± 0.08. Explaining what makes an image poor quality is a difficult task, with inter-rater F1-scores varying between 0.26 and 0.81. Part of the disagreement can be ascribed to personal preference and level of experience with teledermatology, as some dermatologists tend to reject a larger proportion of images than others.

ImageQX reaches dermatologist-level performance on assessing the image quality on all quality assessment classes except for *no skin*. One reason for this lapse may be the low amount of training data for images with no skin. For poor image quality explanations, ImageQX obtains F1-scores within a standard deviation of the inter-rater agreement for all explanations except *blurry*.

Within a real world use case, the high specificity on both the image quality assessment and poor image quality explanation suggests that the image retake burden placed on the users would be low—only truly low-quality or irrelevant images would be flagged for retake. A low percentage of *poor quality*, *no skin*, or *healthy skin* images are likely to be seen by dermatologists. Poor image quality explanations also show high specificity, indicating that, if given proper guidance on how to fix each issue, users would find them useful in their retake attempt. By changing the threshold for poor quality image detection or for the image quality explanations we can further reduce the poor quality images sent to the dermatologists. Such an intervention should be carried out after thorough testing with both patients and dermatologists to ensure that we identify the ideal balance between asking patients to retake the images without being too disruptive.

A Grad-CAM analysis of the poor image quality explanations on an example image shows that ImageQX mostly bases its decisions on relevant areas. The *blurry* attention map is focused on the blurry lesion, whereas *bad light* concentrates on a slightly shaded area to the left of the lesion. *Low resolution* illustrates the debugging capabilities of Grad-CAMs: ImageQX bases its assessment primarily on the background rather than the original image. If these attention maps were to be presented to users alongside the explanations, they could help focus the users' attention to which sections of the image require improvement. For example, the Grad-CAM map for *blurry* suggests that the users should focus on the lesion instead of ensuring that the background is not blurred.

These findings open up several exploration avenues. First, by adding more nonskin images from publicly available datasets we could improve the *no skin* performance. This dataset addition requires the data to be from the same distribution, that is, smartphone images, to avoid in-class domain shift. Second, to more accurately model the uncertainty inherent in the image quality assessment task, we could train ImageQX using soft labels. Third, we believe that by introducing a skin segmentation network as preprocessing we would avoid misclassifications because of ImageQX focusing on the background. One drawback of this approach is the failure case of the segmentation network: if the segmentation removes the areas containing skin, the image quality assessment classifier is bound to fail. Finally, we would like to perform a usability study to quantify the impact an on-device image quality assessment network would have on the time to diagnosis and treatment in a teledermatology setting. Such a study would require an in-depth analysis of how to best communicate the image quality assessments and explanations to the patients.

## Conclusions

Our work on ImageQX introduced several elements of novelty. First, we quantified the dermatologist levels of agreement on what constitutes a high-quality image for a teledermatological consultation and their reasoning when tagging images as low quality. Second, we introduced ImageQX, an expert-level image quality assessor that explains its reasoning for marking an image as poor quality. The added explainability component aims to facilitate the patient understanding on how to improve images. Moreover, with a size of only 15 MB, ImageQX can be easily packaged and deployed in a teledermatology mobile application, and thus incorporated as a step between users taking photos and sending them. Having such a network integrated in the application during the data collection step of this study would have prevented 1,819 *poor quality* or *no skin* images from being sent for assessment to the dermatologists. In the future, we will perform a validation study to quantify the impact of introducing such a method within a consumer facing teledermatology setting.

Our solution offers an improvement to the current consumer facing teledermatology flow by increasing the likelihood that patients send better photos, decreasing the time spent by dermatologists on diagnosing a single patient, and reducing the time needed to arrive at a diagnosis and a treatment for patients.
